# The reliability of nonlinear least-squares algorithm for data analysis of neural response activity during sinusoidal rotational stimulation in semicircular canal neurons

**DOI:** 10.1371/journal.pone.0190596

**Published:** 2018-01-05

**Authors:** Pengyu Ren, Bowen Li, Shiyao Dong, Lin Chen, Yuelin Zhang

**Affiliations:** 1 Department of Neurosurgery, Xi’an Jiaotong University School of Medicine, Xi’an, People’s Republic of China; 2 Departments of Otolaryngology-Head & Neck Surgery, Johns Hopkins University School of Medicine, Baltimore, Maryland, United States of America; 3 Division of Health Sciences Informatics, Johns Hopkins University School of Medicine, Baltimore, Maryland, United States of America; 4 Department of Biomedical Engineering, Johns Hopkins University School of Medicine, Baltimore, Maryland, United States of America; 5 Department of Radiology and Radiological Science, Johns Hopkins University School of Medicine, Baltimore, Maryland, United States of America; Lanzhou University of Technology, CHINA

## Abstract

Although many mathematical methods were used to analyze the neural activity under sinusoidal stimulation within linear response range in vestibular system, the reliabilities of these methods are still not reported, especially in nonlinear response range. Here we chose nonlinear least-squares algorithm (NLSA) with sinusoidal model to analyze the neural response of semicircular canal neurons (SCNs) during sinusoidal rotational stimulation (SRS) over a nonlinear response range. Our aim was to acquire a reliable mathematical method for data analysis under SRS in vestibular system. Our data indicated that the reliability of this method in an entire SCNs population was quite satisfactory. However, the reliability was strongly negatively depended on the neural discharge regularity. In addition, stimulation parameters were the vital impact factors influencing the reliability. The frequency had a significant negative effect but the amplitude had a conspicuous positive effect on the reliability. Thus, NLSA with sinusoidal model resulted a reliable mathematical tool for data analysis of neural response activity under SRS in vestibular system and more suitable for those under the stimulation with low frequency but high amplitude, suggesting that this method can be used in nonlinear response range. This method broke out of the restriction of neural activity analysis under nonlinear response range and provided a solid foundation for future study in nonlinear response range in vestibular system.

## Introduction

The vestibular sensory system is termed the “sixth sense” and plays a vital role in daily life by contributing to visual stabilization [1–3], head and body posture maintenance [4–6], cognition [7–9], spatial orientation construction [10] and navigation [11]. Efficient processing of vestibular neurons input is critical for an animal’s survival. Therefore, a good understanding on how vestibular sensory pathways encode and transmit information to the upstream neurons and brain under various conditions is a major goal in neuroscience. Generally, neural response property and information transformation, the basics of a sensory pathway, are explored through the activities of each neuron in response to stimulation or downstream neural inputs. Therefore, a reliable mathematical method describing neural response under stimulation is extremely vital to analyze the response properties and strategies of information decoding and encoding in neurons at each stage within a hierarchical system.

Over the past 50 years, the neural processes of the vestibular sensory system responding to outside stimulations have been studied through sinusoidal systems analysis tools and methods [12–18], because the free head motions in daily life were similar to sinusoidal motions in most of the situations. Within these analysis tools and methods, least-squares regression analysis [19], Fourier analysis [13], Discrete Fourier transforms [14], Levenberg-Marquardt methods [17,18], maximum likelihood estimation method [20] and hierarchical least squares methods [21] were chosen, aiming to describe the dynamic neural response activity in linear response range during sinusoidal stimulation. However, although these methods are widely used for data analysis of neural response activity in vestibular system, there is no study reporting the reliability of these methods. Additionally, most of the previous studies using these methods for data analysis were under the data inclusion criteria of low head angular velocity, low linear acceleration and no neural activity silence (cutoff) during inhibitory stimulation [12–14,17–19,22]. These criteria restrict the neural response activity within a narrow linear response range and limit our understanding regarding the properties underlying more widely nonlinear response range. Furthermore, the peculiar characteristics of the vestibular system also make us concern about the reliability of these methods: the response-intensity and information encoding between excitatory and inhibitory stimulation within the same magnitude are asymmetric [14,17,23–25], especially significant under large magnitude [19,22,26–29]. Considering these problems above, these data analysis methods may encounter challenge, especially in nonlinear response range in vestibular system.

In order to acquire a reliable method for data analysis of neural response under sinusoidal stimulation in the vestibular system, we chose nonlinear least-squares algorithm with sinusoidal model (NLSA) and verified its reliability on describing SCNs response activity during head SRS in an extended nonlinear neural response range. NLSA is a mathematical optimization technique, which can be used to find the optimum matching function for a set of observed data with a model that is nonlinear in unknown parameters trough minimized error of sum of squares [30]. The idea is to approximate the model by a linear one and to refine the parameters by successive iterations [31]. Finally, the coefficient determination (R^2^) providing a measure of how well observed outcomes are replicated by the model [32], is used to assess how reliable each optimized fitting function is to describe the neural response activity under a special stimulation.

## Materials and methods

### Animals

In the present study, a total of 30 adult female chinchillas (*C*. *laniger*), 480–550 g body weight, were housed in groups of two per metallic cage with *ad libitum* access to food and tap water. All the animal surgeries and single unit recording procedures were approved by the Johns Hopkins University Animal Care and Use Committee, and in compliance with the National Institutes of Health guide for the care and use of Laboratory animals.

### Orientation assessment

A coordinate system to assess the orientation of animal head and each semicircular canal in three-dimensional space was constructed, as reported by previous studies [13,14]. In this coordinate system, the XY-plane was always paralleled to the horizontal plane, the XZ-plane and the YZ-plane were perpendicular to the XY-plane, and also vertical to each other. The animal head was fixed in a stereotaxic platform mounted within a gimbal atop a servo-control earth-vertical rotating table (Kollmorgen goldline direct drive rotary servo motor, model D083M-22-1310) whose rotation plane was paralleled to the horizontal plane. Therefore, the orientation of the animal head could be freely adjusted. The original orientation of the animal head for the angular measurement of three canal planes was the Y-axis perforated from the right external auditory canal to the left of two ears, the X-axis perforated from the center of the connection-line between left and right external auditory canals to the posterior edge of the incisors meeting and entering the maxillary bone [33]. Therefore, the longitudinal axis of the animal body and the head line connecting centers of left and right external auditory canals both run through the rotation axis.

### Surgery and recording techniques

The surgical approach was similar to previously studies [13,14]. Animals were maintained under general anesthesia by inhaling 1–5% isoflurane [34] before and during surgery and single unit recording. Vestibular nerves and Scarpa’s ganglion were exposed approximately at 0.5–1.0 mm anterior to the facial nerve canal and 1.0–2.0 mm medial to the superior ampulla through extracranial approach.

To record neural activity, glass micropipette electrode (WPI, model M1B100F-4) was pulled and afterward filled with 3M NaCl solution to achieve 20–40 mΩ impedance. Then, the glass electrode was held in position over the nerve using a three-dimensional manipulator (You, model US-3F) fixed on a hydraulic microdrive (Narishige International USA, models MO-22). Next, the glass electrode driven by the microdrive was carefully inserted into the vestibular nerve. Then the microdrive was slowly advanced until the glass electrode isolated a single nerve fiber or neuron cell body and identified extra axonal activity of neuron (spikes). Neuron signals were inputted into an extracellular amplifier (Dagan, model 2400A) at a gain from 500 to 5,000 and the band-pass filter from 300 to 3,000 Hz. Finally, neural activities were recorded by the CED Spike2 neural signal acquisition software.

### SCNs innervation identification and canal plane adjustment

Once a neuron was well isolated, a combination of stimuli consisting of yaw and pitch animal head rotation by hand were performed, and afterwards the innervating semicircular canal was immediately identified through monitoring the response activity during rotations [13]. Then the platform was adjusted to make sure that the plane of the identified semicircular canal was brought into the rotation plane according to Hullar’s measure [33]. In this situation, the spontaneous activity of each neuron was recorded at least 20 seconds before the start of the stimulation. To describe a sinusoidal stimulation, two parameters are needed, frequency and amplitude. Thus, frequency and amplitude test were designed for SCNs to investigate the reliability of NLSA.

### Neural response activity in response to frequency

Neural activity in response to frequency was measured through constant amplitude (80 deg/s) SRS applying multiple frequencies. For a complete frequency trial to a neuron, all three sequentially increased frequencies (0.2, 0.5 and 1 Hz) were applied in this test. The stimulus with any frequency was repeated 10–20 cycles.

### Neural response activity in response to amplitude

Neural activity in response to amplitude was tested through constant frequency (0.2 Hz) SRS applying sequentially increased amplitudes (peak head angular velocity). A complete amplitude trial to a neuron must include all the 6 amplitudes (60, 80, 100, 120, 150, 180 deg/s). Additionally the stimulus with any amplitude was repeated 10–20 cycles.

### Nonlinear least-squares algorithm with sinusoidal model

The nonlinear least-squares algorithm is a mathematical method for parameters estimation of static nonlinear model, where the minimum of error sum of squares is the criterion [30]. In the present study, the static nonlinear model was a sinusoidal model and possessed the following form:

y=Asin(ωx+φ)+B (1)

where *x* was the given system input, *y* was the observed system output, *A* was the amplitude, *ω* was the frequency, *φ* was the phase and *B* was the offset. When a set of *n* data points, (*x*_1_, *y*_1_), (*x*_2_, *y*_2_), ⋯, (*x*_*n*_, *y*_*n*_), were observed during the system test, the parameters, *A*, *ω*, *φ* and *B*, were calculated. The amplitude *A* and offset *B* could be acquired straightly from the measurement of the range of *y*_1_ to *y_n_* in raw data, the ordinary frequency *ω* was already known as the stimulation frequency (SRS). Therefore, only the last parameter, *φ*, needed to be estimated through the following equation based on these data:

$$S=\min\sum_{i=1}^{n}{\left[y_{i}-\left(A\sin\left(\omega x_{i}+\varphi \right)+B\right)\right]}^{2}$$ (2)

where *S* was the least error sum of squares, and 1 ≤ *i* ≤ *n*. Then, the lower and upper bound were set for the starting value of the phase. The algorithm for sinusoidal fitting was implemented through function lsqnonlin in Matlab (version 9.2.0, MathWorks, Natick, MA). After each time of fitting, the returned phase value (*φ*) was used as the starting point for the next iteration of fitting until the standard deviation was under the threshold set before the experiment.

### Data analysis

All data were imported into Matlab (version 9.2.0, MathWorks, Natick MA) to calculate mean spontaneous discharge rate (MSDR, spikes/s), interspike interval (ISI, ms), standard deviation (SD) of ISI, coefficient of variation (CV) and normalized coefficient of variation (CV*). Based on the regularity of neural discharge activities in resting state, SCNs were classified into different groups. The regularity was quantified by CV* and calculated from the distribution of ISI within a section of spontaneous activity of 20 seconds. SD was indicated by σ_ISI_, ISI mean was symbolized by μ_ISI_, and CV was defined as CV = σ_ISI_ / μ_ISI_, which could be straightly used to show the discharge regularity of a neuron. However, CV varied with the mean of ISI [27], thus CV*, when ISI mean was normalized to 15 ms, was chosen to quantify the discharge regularity and classify SCNs into regular (CV* < 0.10) and irregular group (CV* > 0.10) [14,29,35] in the present study. To address the reliability, NLSA was used to fit both neural response activity data and stimulation with sinusoidal function within each rotation section. Only the neural response activity to SRS for more than 5 complete cycles was collected for further analysis (Data inclusion criteria). Then R^2^ was used to assess how reliable each fitting function was to describe the neural response activity, which ranged from 0 to 1. The more reliable the fitting function, the more approximated R^2^ to 1.

### Statistical analysis

Data were presented as mean ± SD. Student’s *t*-test was used to determine whether the average results of two groups differed significantly from each other. The repeated measures ANOVA (RANOVA) was used to compare the different results among different frequencies and amplitudes (more than two groups) within the paired neuron response.

## Results

262 SCNs were recorded, divided into 152 horizontal, 75 anterior and 35 posterior SCNs. According to the discharge regularities in resting state, 189 neurons were classified into regular SCNs and other 73 neurons were irregular SCNs. Under the data inclusion criteria (see Methods), a number of 265 and 182 fittings were implemented on regular and irregular SCNs separately based on different stimulation.

### Description of neural response activity and fitting function

**Fig 1:**
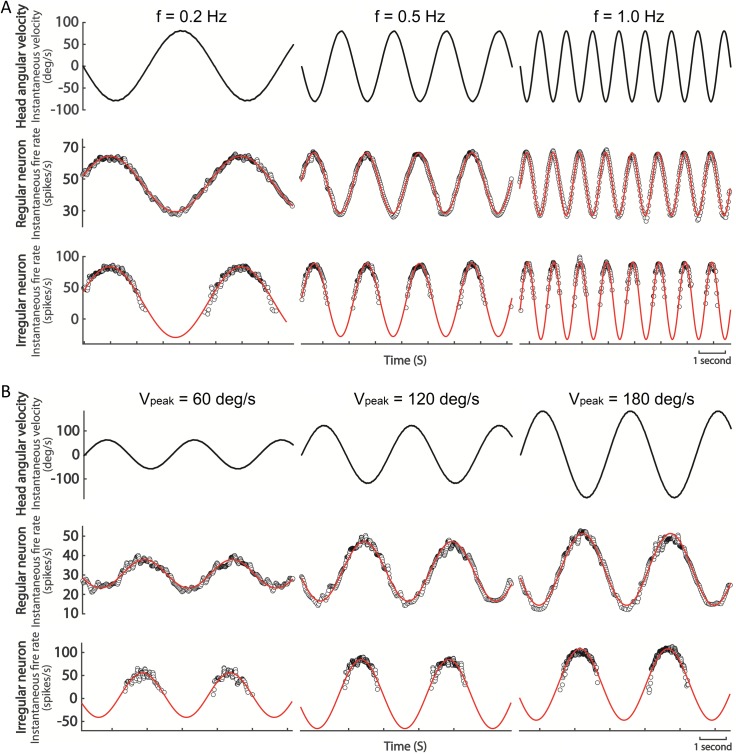
**Description of neural response activity and fitting functions in frequency test (A) and amplitude test (B).** The upper black solid curves indicated the dynamic head instantaneous angular velocity. The black circles in the medium and bottom rows show the dynamic instantaneous neural response activity (instantaneous fire rate) to the corresponding stimulation for regular and irregular SCNs separately. The red solid curves over the data of neural response activity indicate the fitting function calculated by nonlinear least-squares algorithm with sinusoidal model.

The goal of the present study was to evaluate how reliable the neural response activity can be when described by NLSA with sinusoidal model. According to the experimental protocol used, SCNs were tested by frequency test or amplitude test and some examples are shown in Fig 1. Fig 1A shows examples of a regular SCN (medium, CV* = 0.024) and an irregular SCN (bottom, CV* = 0.127) responding to SRS in frequency test (0.2, 0.5 and 1.0 Hz). All the fitting functions (red solid curves) described the dynamic neural response activity quite precisely during SRS. R^2^ values for each fitting function also confirmed the above results: R^2^ > 0.98 for regular and R^2^ > 0.91 for irregular. Fig 1B exhibits examples of a regular SCN (medium, CV* = 0.036) and an irregular SCN (bottom, CV* = 0.364) responding to SRS in amplitude test (we just chose 60, 120 and 180 deg/s as examples). All the fitting curves were also well qualified to describe the corresponding neural response activity data (R^2^ > 0.85 for regular and R^2^ > 0.67 for irregular).

### NLSA reliability assessment in SCNs

**Fig 2:**
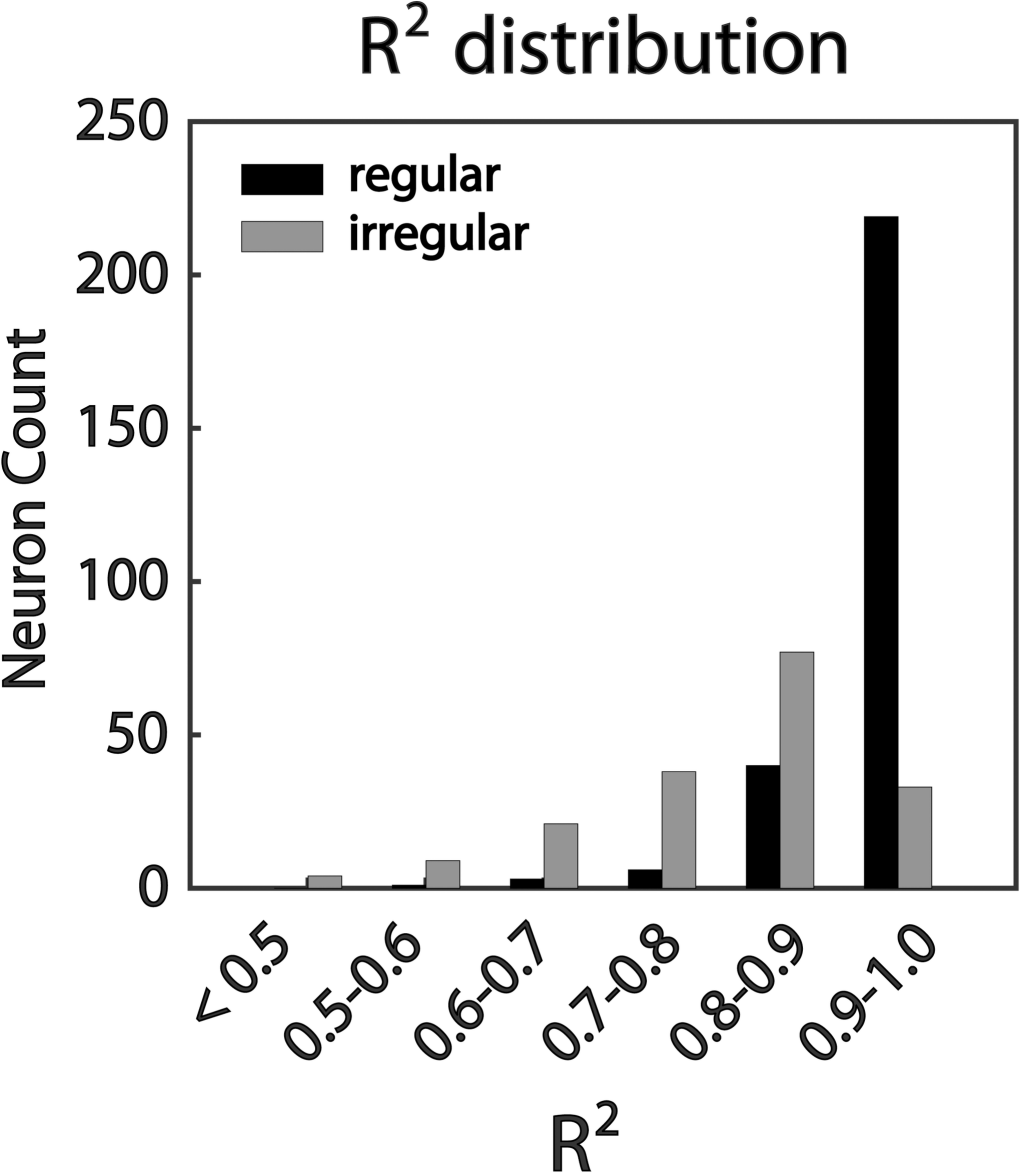
R^2^ distribution in the entire SCNs population. 256 and 182 fittings were implemented on regular and irregular SCNs separately. Most of R^2^ of the fitting function calculated by NLSA with sinusoidal model aggregated within high value range (0.7–1.0). Furthermore, the R^2^ in regular group was significantly higher than that in irregular group (*t*-test, *P* < 0.001).

Fig 2 shows the R^2^ distribution of all fitting functions based on neural response activity data recorded under different stimulation. R^2^ in the entire SCNs population was quite satisfactory, especially for regular neurons. In the regular group, a percentage of 81.1 neurons showed an R^2^ larger than 0.9 and 96.2% were above 0.8. Although the R^2^ in the irregular group was not as well as that in the regular group, it was still good. A percentage of 60.4 irregular SCNs showed an R^2^ larger than 0.8, and more than 81.3% of irregular SCNs showed an R^2^ above 0.7. Therefore, most of R^2^ aggregated in the high value range (0.7–1.0) although the stimulation variation was particularly large. As a conclusion, NLSA with sinusoidal model was qualified to describe the neural response activity during SRS in a wide response range.

### Effect of CV* on reliability

**Fig 3:**
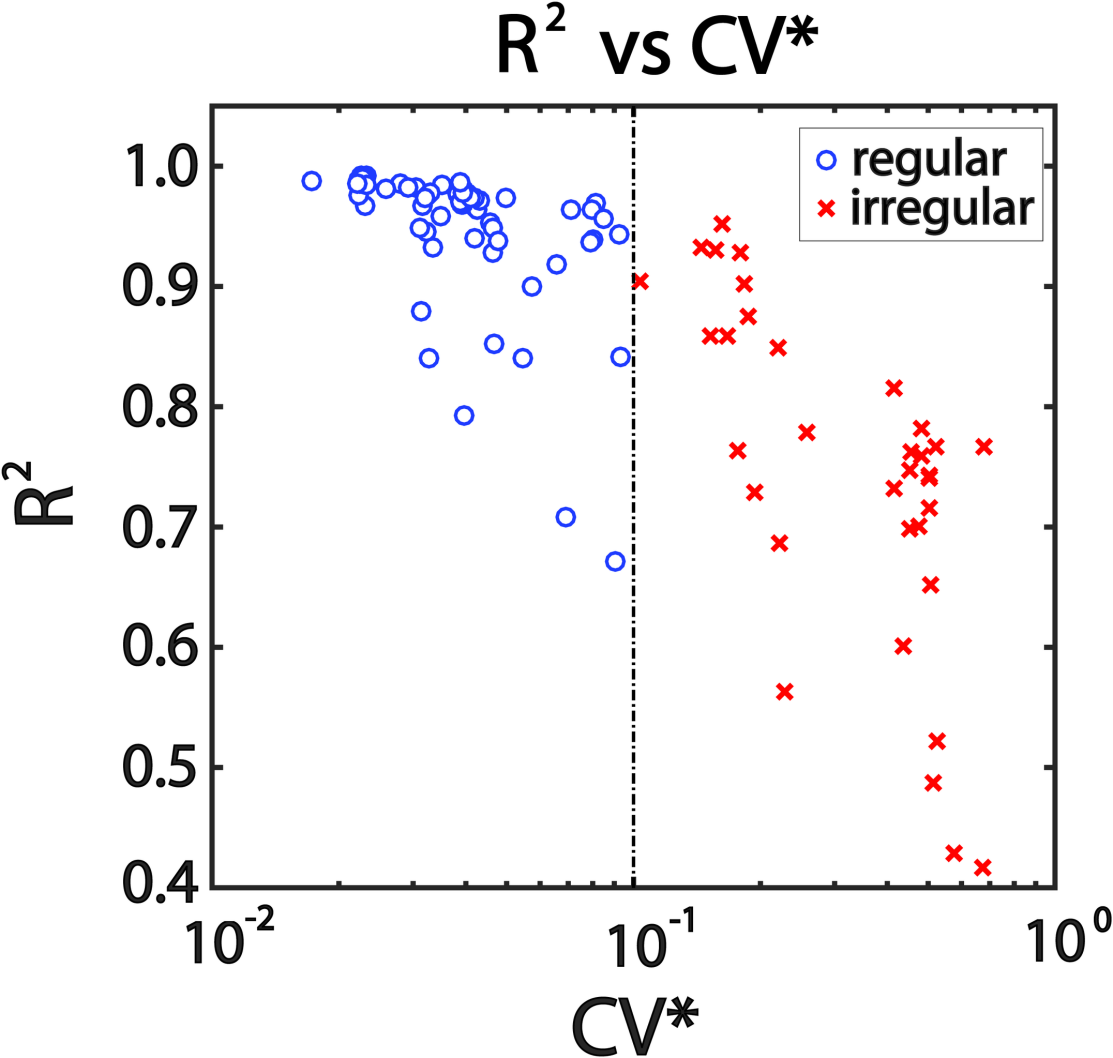
Effect of discharge regularity on the reliability of NLSA (n = 98). R^2^ of fitting function based on the data of neural response activity under constant SRS (frequency = 0.5 Hz, amplitude = 80 deg/s) as a function of CV*. The more regular the discharge regularity (quantified by CV*), the more reliable the NLSA.

In order to address the impact factors associated to NLSA reliability and further extend the application range, we tested R^2^ with some potential related factors. Since we noticed that R^2^ of regular neurons tended to dominate a higher value range than that of irregular, as shown by Fig 2, CV* was the first impact factor to be investigated. All the data of neuron response activity to 0.5 Hz SRS with 80 deg/s were collected, and then a plot for R^2^ as a function of CV* was illustrated in Fig 3. R^2^average value for regular SCNs (0.95 ± 0.06) was higher than that for irregular (0.75 ± 0.14), and the difference was significant (*t*-test, *P* = 0.004). Further, R^2^ exhibited a strong dependence on discharge regularity quantified by CV*: the more irregular (larger CV*) the discharge regularity, the smaller the value of R^2^. In conclusion, R^2^ of the fitting function for neural response activity description has a negative correlation with CV*.

### Effects of frequency and amplitude on reliability

**Fig 4:**
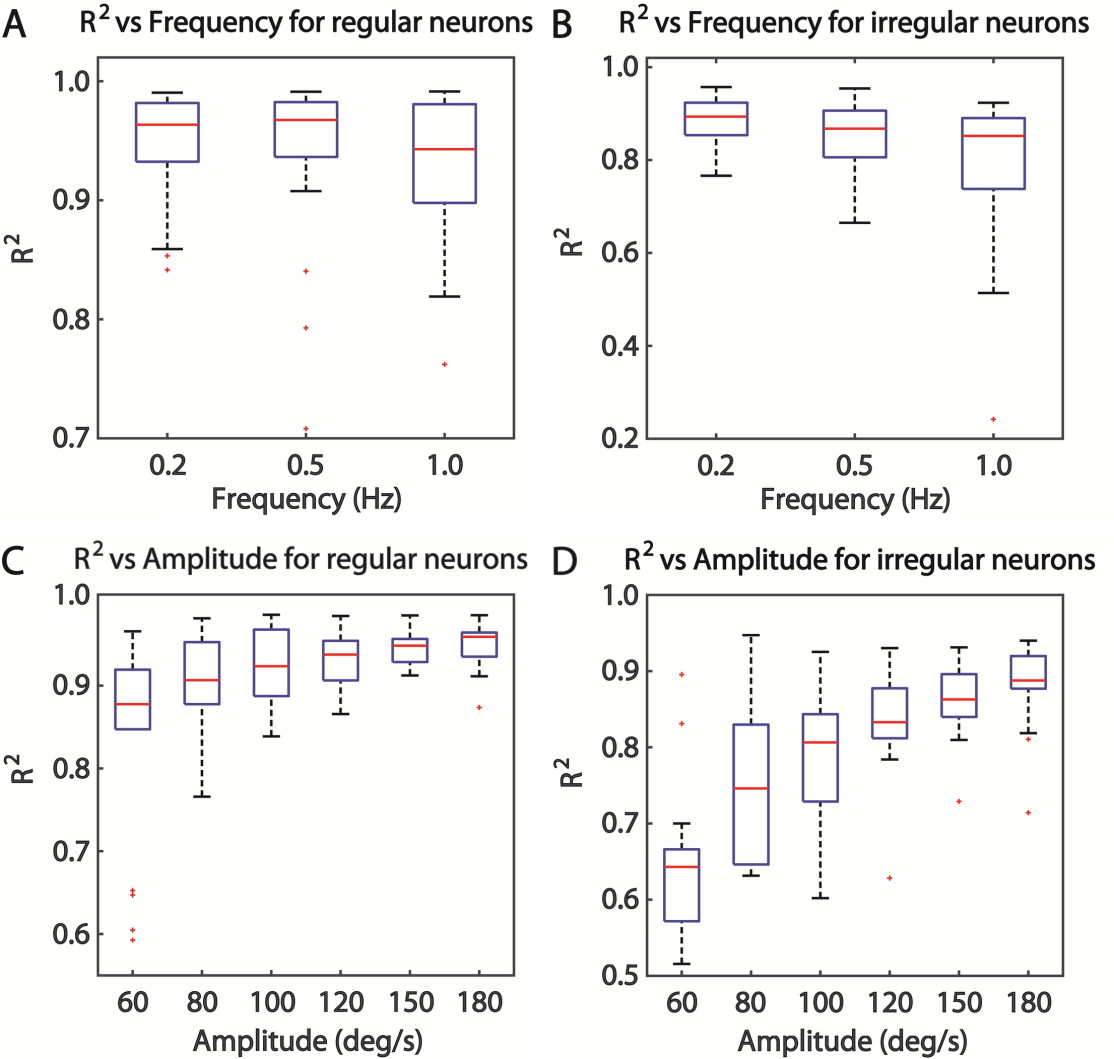
Effects of stimulation on NLSA reliability. A (n = 30) and B (n = 20) exhibited the negative effect of frequency on the reliability for data analysis of regular and irregular SCNs separately. C (n = 18) and D (n = 18) showed the positive effect of amplitude on the reliability for data analysis of regular and irregular SCNs respectively.

Generally, the modulation of neural response activity can be driven by stimulation. Thus, SRS frequency and amplitude (peak head velocity) are other potential impact factors. First, we investigated the relationship between R^2^ and frequency based on the data of SCNs completing the complete frequency trial, and then R^2^ was plotted as a function of frequency for regular and irregular neurons in Fig 4A and 4B respectively. Although R^2^ values changed slightly following the increased frequency both in regular and irregular group, statistical analysis exhibited a significant negative effect of frequency on R^2^ from 0.2 to 1.0 Hz (RANOVA, *P* = 0.001), which indicated that increased frequency reduced the reliability of this method for neural response activity description. Additionally, this negative effect was stronger in irregular group than that in regular (RANOVA, *P* = 0.028). Second, we examined the effect of amplitude on reliability of NLSA based on neural response activity data acquired from complete amplitude trial, which is shown in Fig 4C and 4D. R^2^ increased continuously as amplitude increased from 60 to 180 deg/s, which was more conspicuous in irregular group. Statistical analysis further confirmed this conclusion of a significant positive effect of amplitude on R^2^ within this range (RANOVA, *P* < 0.001), which indicated that large amplitude could improve the reliability of NLSA for neural response activity description. Furthermore, the positive effect of amplitude on irregular group was stronger than that on regular (RANOVA, *P* = 0.002). Additionally, R^2^ distribution range (variation) was reduced by increased amplitude in both regular and irregular group (Fig 4C and 4D), which was the opposite as in frequency analysis (Fig 4A and 4B). All the above results implied that NLSA with sinusoidal model was qualified to analyze neural response activity data in a wide response range but not suitable for the data analysis under extreme high frequency.

## Discussion

Exploration of neural response properties and neural information transformation through neural activity under stimulation is a main work in neuroscience. Therefore, a reliable mathematical method for data analysis of neural response activity is extremely vital.

In the present study, we reported for the first time the reliability of NLSA with sinusoidal model on the description of neural response activity during SRS in the vestibular system. Our data showed that R^2^ of the fitting function in the entire population of SCNs were quite satisfactory, more than 96.6% of regular SCNs had an R^2^ above 0.8 and more than 81.3% of irregular was above 0.7, suggesting that NLSA was a reliable mathematical tool and could be used to analyze the data of neural response activity under SRS.

Furthermore, current study investigated for the first time the reliability variation of a mathematical method under different situations, which could guide us to use the analysis method correctly and finally acquire reliable results. In most previous studies [12–14,35], a mathematical method was usually chosen to analyze the neural response activity within a wide response range ignoring the reliability variation, usually leading us to ignore the reliability of the final results. Thus, it was necessary to investigate the potential reliability impact factors for NLSA with sinusoidal model. Here we demonstrated that not only discharge regularity (internal factor) but also stimulation (external factor) could impact the reliability of this analysis method. The effect of discharge regularity on reliability was significantly negative, suggesting that this method was more qualified to analyze the data of regular SCNs. This conclusion is in accordance to previous findings on larger variation of neural electrophysiological activity in more irregular neuron [14,29,35]. The effect of frequency and amplitude on reliability was negative and positive respectively, which indicated that this method was more suitable for the data analysis of neural response activity under rotational stimuli with low frequency and high amplitude. These conclusions are very important for the application range selection of this mathematical method.

It is also the first time that the reliability of a mathematical method was revealed within nonlinear neural response range [19] in the vestibular system. Although within linear response range, vestibular neurons have been characterized in terms of dynamic response property from relatively low to extreme high frequency [12–14,35], neural response detection threshold [17,18], preferred rotation plane in three-dimension space [33,36], and linear information encoding for upstream neurons or brain [13,15,16,37–39], we still don’t have enough information regarding neural response property in the broader nonlinear response range because of lacking a reliable method for data analysis of asymmetric neural response activities between excitatory and inhibitory stimulation under the same stimulation intensity. Therefore, a reliable method is urgently needed in nonlinear response range. Our current work confirmed that NLSA with sinusoidal model was more qualified to analyze the data under stimulation with higher amplitude (peak angular velocity), indicating that it could be used in nonlinear response range, helping us to explore the undiscovered neural response properties and information transformation in nonlinear response range.

Overall, NLSA with sinusoidal model resulted to be a pretty reliable mathematical method to analyze neural activity in response to SRS in SCNs. This mathematical method was more reliable for the data analysis of regular neurons and neural response activity under low frequency stimulation in nonlinear response range. All these conclusions reduced the previous restrictions and extended the application into nonlinear response range, providing a solid foundation for our further study in vestibular system. However, this just creates a new beginning for the reliability discussion of mathematical methods used in neural activity analysis. Further studies are needed in the future to compare the results of NLSA to other mathematical methods, for revealing more accurate characteristics of neuron system based on superior analysis method selection.

## Supporting information

S1 File The original data sets for Fig 2. DOI: 10.6084/m9.figshare.5732340.
(XLSX)

S2 File The original data sets for Fig 3. DOI: 10.6084/m9.figshare.5732346.
(XLSX)

S3 File The original data sets for Fig 4. DOI: 10.6084/m9.figshare.5732349.
(XLSX)

S4 File All original data sets associated with the present study. DOI: 10.6084/m9.figshare.5732352.
(XLSX)
